# B1b Cells Recognize Protective Antigens after Natural Infection and Vaccination

**DOI:** 10.3389/fimmu.2014.00535

**Published:** 2014-10-31

**Authors:** Adam F. Cunningham, Adriana Flores-Langarica, Saeeda Bobat, Carmen C. Dominguez Medina, Charlotte N. L. Cook, Ewan A. Ross, Constantino Lopez-Macias, Ian R. Henderson

**Affiliations:** ^1^MRC Centre for Immune Regulation, Institute for Microbiology and Infection, School of Immunity and Infection, Institute for Biomedical Research, Medical School, University of Birmingham, Birmingham, UK; ^2^Medical Research Unit on Immunochemistry, National Medical Centre “Siglo XXI”, Specialties Hospital, Mexican Institute for Social Security (IMSS), Mexico City, Mexico

**Keywords:** B1b cells, antibody responses, bacterial infections, vaccines, B-cells

## Abstract

There are multiple, distinct B-cell populations in human beings and other animals such as mice. In the latter species, there is a well-characterized subset of B-cells known as B1 cells, which are enriched in peripheral sites such as the peritoneal cavity but are rare in the blood. B1 cells can be further subdivided into B1a and B1b subsets. There may be additional B1 subsets, though it is unclear if these are distinct populations or stages in the developmental process to become mature B1a and B1b cells. A limitation in understanding B1 subsets is the relative paucity of specific surface markers. In contrast to mice, the existence of B1 cells in human beings is controversial and more studies are needed to investigate the nature of these enigmatic cells. Examples of B1b antigens include pneumococcal polysaccharide and the Vi antigen from *Salmonella* Typhi, both used routinely as vaccines in human beings and experimental antigens such as haptenated-Ficoll. In addition to inducing classical T-dependent responses some proteins are B1b antigens and can induce T-independent (TI) immunity, examples include factor H binding protein from *Borrelia hermsii* and porins from *Salmonella*. Therefore, B1b antigens can be proteinaceous or non-proteinaceous, induce TI responses, memory, and immunity, they exist in a diverse range of pathogenic bacteria, and a single species can contain multiple B1b antigens. An unexpected benefit to studying B1b cells is that they appear to have a propensity to recognize protective antigens in bacteria. This suggests that studying B1b cells may be rewarding for vaccine design as immunoprophylactic and immunotherapeutic interventions become more important due to the decreasing efficacy of small molecule antimicrobials.

## The Importance of B-Cells in Vaccination

B-cells carry a unique signature through the expression of a distinct surface immunoglobulin receptor. Under favorable circumstances, if the cognate antigen is engaged and appropriate secondary signals are received, the B-cell can differentiate into a plasma cell and secrete antibody through at least two pathways (1, 2). Depending upon the specificity, antibody can help prevent infections from establishing and protecting against a number of infectious diseases. B-cells can have additional activities during infection, which are not necessarily related to antibody production, where they provide signals through contact-dependent and -independent mechanisms as seen during experimental infections with the helminth *Nippostrongylus brasiliensis* (3). Such antibody-independent activities of B-cells are clearly important in infectious and non-infectious diseases (4–6). Thus, B-cells are important modulators of the host response and the growing and extending interest in the effector activities of B-cells is a welcome expansion of our understanding of the activities of this cell type.

Nevertheless, it is the antibody-mediated effector functions of B-cells that are estimated to save >2 million lives yearly (7). Antibody is behind the elimination of smallpox and the drastic reductions in the prevalence of measles, polio, diphtheria, tetanus, and a plethora of other infections for which vaccines exist, bringing tremendous economic and social benefits (8). Moreover, once infections have been encountered and natural immunity acquired, then the levels of antibody often correlate to the levels of protection against reinfection (9). Vaccines and antibody typically protect at the first encounter with a pathogen, usually before clinical signs are apparent and when bacterial numbers are at their lowest. In contrast, antibiotics are used when bacterial burdens are toward their peak and when clinical signs are more prominent. This game of numbers is probably a key reason why antimicrobial resistance is more common than resistance to a vaccine. As we head toward an era where increased resistance means existing antimicrobials will be less efficacious, there will be an increasing reliance on antibody-mediated mechanisms to protect us. To achieve this requires an efficient way to identify protective antigens. This is an important concept as separating out which antigens are protective from those antigens which are not is a timely, complex, and costly process (10). Therefore, understanding how to efficiently identify protective antigenic targets on pathogens will be a valuable tool for the future control of infection. We propose that understanding the nature and targets of B1 cells, particularly B1b cells, is one such route for this. In this review, we discuss elements associated with B1 cells and infection, with a major emphasis on the relationship between bacterial antigens and B1b cells. This is in part to maintain a focus in the review, but also because other elements of B1 cell biology, particularly B1a cell biology, such as their development, role in housekeeping functions, and in diseases, such as autoimmunity, have been elegantly reviewed elsewhere in detail (11–36).

## The Role of Antibody in Infections and Responses to Vaccination

Virtually, all vaccines work through the induction of antibody. The key point here is that, in general, antibody needs to be pre-existing at the time of pathogen encounter indicating the importance of inducing a persisting plasma-cell response to maintain this protective blanket of antibody. It is clearly desirable to induce B-cell memory to complement these activities and to augment antibody levels after antigen re-encounter, but responses to vaccination with T-independent (TI) antigens such as purified capsular polysaccharides show that robust memory is not essential for vaccines to work (37). Antibody induced to T-dependent (TD) antigens, such as proteins, is induced in two waves. Initially, after antigen encounter extrafollicular (EF) IgM is induced, which is typically of modest affinity as at the earliest time after antigen encounter, it is not derived from germinal centers (GCs; see below). Slightly later, the first IgG is detected, which increases in affinity with time as the GC makes a greater contribution (1, 2). Nevertheless, IgM is normally present with IgG to make a significant contribution to protection (38–42). In mice, the isotype of IgG induced can reflect the nature of the immune response. IgG3 is the dominant switched isotype after TI antigens, whereas IgG1 and IgG2a reflect T helper (Th) 2 and Th1 responses, respectively (38). *In vivo*, the predominant IgG isotype to a single antigen can vary depending upon the antigenic context in which it is encountered by the immune system (43). A relationship between the direction of Th responses and the direction of IgG switching is less clear in human beings, although some responses are more associated with certain IgG isotypes, for instance IgG2 and IgG4 antibodies are commonly found to LPS O chain and helminths, respectively (44, 45).

Multiple experimental models of infection show that IgM is critical for much of the short- and long-term protection afforded after natural infection and that the functional roles of IgM and IgG are likely to be synergistic (40, 46). The high avidity of pentameric IgM means that it is efficient at activating complement, whereas not all IgG isotypes are equally efficient at doing this (47). In response to most infections or proteinaceous vaccines, IgG titers will rise over many weeks, whereas IgM titers typically remain steady or fall. High affinity IgG is not induced by purified capsular polysaccharides (48).

For instance, the value of IgG has been demonstrated in studies using antibody generated during a natural non-typhoidal *Salmonella* infection or by an experimental protein vaccine against this infection that can induce TI and TD responses (46, 49). In these studies, the consistent observation was that IgG could account for up to 95% of the protection observed in wild-type (WT) mice, although surprisingly the additional benefit of IgG was not necessarily related to it being of high affinity. However, the role of IgG in the absence of IgM was not assessed in these studies. An additional consideration is that cell-free, antibody-dependent, complement-mediated bactericidal killing in mice may not be equally active against all infections. This is notable in *Salmonella* infections, where mouse serum is not effective at killing the organisms in bactericidal assays *in vitro* as human serum is, and so the true value of IgM and antibody *per se* may be under-represented in murine systems (50). A further consideration is that the amount of antibody induced to a single antigen can affect outcome. Individuals with HIV/AIDS have a known increased susceptibility to invasive non-typhoidal *Salmonella* infections (51). The reason for this was recently attributed to elevated titers of inhibitory IgG to LPS and removal of this antibody enabled bacterial killing by the remaining non-inhibitory antibodies (52). A similar observation has been made in patients with bronchiectasis and a *Pseudomonas aeruginosa* infection (53). A proportion of these patients have markedly elevated titers of IgG2 to LPS O chain that inhibits cell-free or cell-dependent killing of bacteria. Therefore, efficient protection after infection or vaccination requires antibody to selective targets, present in the right amount and of the right isotype.

## The Development of Antibody Responses to T-Dependent and Independent Antigens

There are classically three types of antigens (54–56). Some, such as LPS, can act as TI type I antigens, which induce specific and non-specific antibody responses through direct stimulation of the B-cell. A second class of antigens is TI type II antigens. Antigens that fall into this group include purified capsular polysaccharide vaccines such as those generated from pneumococcus and the experimental antigen, haptenated Ficoll. Typical haptens include 4-hydroxy-3-nitrophenyl acetyl (NP) and variations of this such as DNP and TNP. A feature of TI–II antigens is that within a single molecule, there are multiple repeats of the same epitope. This means that there is spatial co-localization of the same epitope and when a B-cell encounters the antigen, then multiple surface B-cell receptors are engaged in parallel (57). This drives a strong signaling response within the cell and abrogates the necessity for T-cell support to generate an antibody response. The third class of antigens is TD antigens, which are generally proteins. This is then processed and presented to T-cells via MHCII to enable their recruitment into the response. Conjugation of a TI–II antigen to a protein carrier can convert it into a TD antigen, a process that typically requires physical linkage (58, 59). The consequences of T-cell involvement in the generation of antibody responses are dramatic, since it ultimately results in the induction of productive GCs and the generation of greater amounts of switched antibody of a higher affinity and the generation of robust long lived and memory B-cell responses. Furthermore, conjugating a TI antigen to a protein carrier enables responses to capsular polysaccharide vaccines to be induced in certain groups, such as infants, in whom responses would otherwise be refractory (60, 61). The concept of classes of antibody responses has been the focus of recent discussion (62). This has led to the development of the idea of a TI–III response, which displays unique features, as well as sharing some with other types of B-cell responses. The distinct features of the TI–III response focus on the support provided by additional bone marrow-derived cells including neutrophils, monocytes, mast cells, and basophils and are induced after infection with bloodborne or gut bacteria. They result in the production of EF antibody and show a marked dependence on TLR signaling for their development.

**Figure 1:**
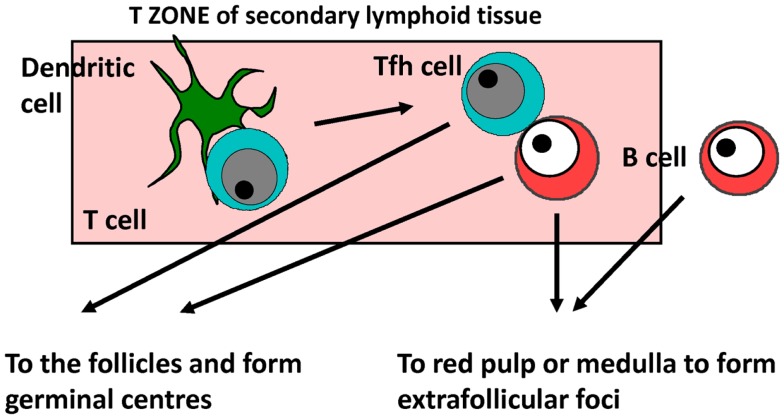
**The development of antibody responses**. Specific antibody responses develop in secondary lymphoid tissues. B-cells responding to T-dependent and T-independent antigens migrate through the T-zone before producing antibody through the germinal center (GC) or extrafollicular (EF) pathway. Tfh cell – T follicular helper.

In mice, responses to model TI-2 and TD alum-precipitated antigens such as NP-chicken gamma globulin or ovalbumin, the kinetics of the immune response has been well characterized for both primary and recall responses and below is a short synopsis based on this non-exhaustive list of references (1, 2, 63–69) (Figure 1). It is likely most of the core features of this response reflect what happens in human beings. After antigen encounter, dendritic cells within the T-zone present and prime T-cells within the first 24 h to enable their rapid differentiation to T follicular helper (Tfh) cells. The dendritic cell subset involved in Tfh priming, Ig switching, and other T effector functions may not always be the same since different subsets can provide these functions in different sites concurrently. For instance, we have found that after flagellin immunization CD103^+^ dendritic cells prime for IgA and IgG in the mesenteric lymph node but not the spleen, and that after *Salmonella* infection monocyte-derived dendritic cells help prime for IFNγ-expression in T-cells but not IgG2a responses (70, 71). In parallel, B-cells that have engaged antigen interact with primed Tfh cells also within the T-zone. Depending upon the signals received, B-cells can essentially have one of the three fates. They can die, or while still in the T-zone start to differentiate and proliferate. A proportion of these cells will migrate to the follicles and with the adequate recruitment of Tfh cells start to form GCs. Other B-cells will migrate to EF sites such as the red pulp of the spleen or the medulla of lymph nodes to generate plasmablasts and ultimately plasma cells. The kinetics are important for this process and so by around 5 days after immunization, an initial wave of short-lived IgM and IgG (predominantly IgG1 in mice) plasma cells is readily detectable in EF sites. These peak by around day 7 before gradually falling over the coming days (72). With slightly delayed kinetics, GCs form within the follicles and contribute the majority of plasma cells by a week or so after immunization. The key points that are applicable to the current discussion are that after proteinaceous molecules there is a parallel development of EF and GC responses, and that there is a robust EF switched IgG response.

What is less appreciated is that the process for the generation of responses to TI–II antigens is similar, but with accelerated kinetics. Thus, after antigen encounter, B-cells still migrate through the T-zone and start to proliferate in this site. Furthermore, some B-cells migrate to the follicles to form GCs that abort around the fourth day after immunization as they lack T-cell help to maintain them. Robust EF plasma-cell responses develop with a rapid expansion of the plasmablast population and the immunoglobulin isotypes most commonly detected are IgM and (in mice) IgG3. There are two major consequences of failing to induce Tfh and GC, mainly the longevity of the antibody response is shorter than that commonly seen to protein antigens, and upon reencountering the same antigen, an accelerated and augmented secondary response is absent. This is a significant clinical problem since hyporesponsiveness to a second immunization with a TI–II vaccine is often seen. Thus, the mechanics of TD and TI antibody responses show many key similarities.

## The Distinct Flavors of B-Cell Subsets

In human beings and mice, there are multiple B-cell subsets, including follicular B-cells and marginal zone (MZ) B-cells, as well as transitional B-cells (17, 31, 73–77). Follicular and MZ B-cells are known as B2 cells. MZ B-cells contain naïve and antigen-experienced B-cells. Follicular B-cells in both species express IgM, IgD and are CD21^int/lo^ and CD23^hi^. The level of diversity in B-cell receptor usage is greatest in follicular B-cells and accounts for most of the enormous antibody diversity seen in B-cells. Their productive engagement in immune responses shows a clear dependence on T-cells. MZ B-cells also share some similarities between mice and human beings in terms of phenotype with both subsets being IgM^+^, IgD^lo/−^, B220^hi^, CD21^hi^, and CD23^lo^. Neither B-cell subset has the capacity to self-renew. There are differences between follicular and MZ B-cells. These differences include the sites where they reside, the more limited antigen repertoire recognized by MZ B-cells, the immaturity of the MZ B-cell compartment after birth, and finally, the capacity of MZ cells to respond to TI–II antigens. These latter two points make the responsiveness of MZ B-cells of significant clinical interest as TI vaccines have poor or negligible efficacy in infants, which correlates with limited MZ B-cell responses.

In mice, there are a number of additional B-cell subsets that are recognized and these come under the umbrella term of B1 cells (17, 22, 23, 26, 28, 31, 33). They are most associated with some serosal sites such as the peritoneal cavity, although they can also be detected in secondary lymphoid tissues and so are probably more widespread than currently appreciated (78, 79). A key feature that distinguishes B1 subsets from B2 subsets is their capacity to self-renew. Thus, transfer of B1 populations into lymphopenic mice will result in their expansion over a period of weeks. This means that it is possible to make B1 chimeric mice by transferring peritoneal cells into a Rag-deficient mouse [e.g., Ref. (80)]. In the steady state in the peritoneal cavity of a WT mouse, about 50% of B-cells are B1 cells, and we find there is an approximate ratio of B1a cells to B1b cells of 2:1 (46, 81). A factor that complicates the study of B1 cells is their more elusive phenotype. They express CD19, low levels of IgD but relatively high levels of IgM. They can express CD43 and CD9, are negative or low expressers for other markers such as CD21 and CD23, and are mostly intermediate for B220 (27, 31, 82–84). In the spleen, many of their characteristics are similar to transitional B-cells when at a transitional T1 stage. Identification of B1a cells is substantially aided by their modest expression of CD5, although in mice expressing CD5 is not necessarily exclusive to B1a cells (85). Another marker closely associated with B1 cells is CD11b, but the frequency of B-cells expressing CD11b can be variable; for instance, during some infections, it alters from that seen during steady-state conditions (46). It is unclear whether the differences in CD11b expression mark distinct B1 subsets or reflect differing developmental stages (86–88). Nonetheless, these factors make examining B1 cells more complicated, particularly in secondary lymphoid tissues, and the often necessary reliance on CD11b as a positive marker, though understandable, is likely to mean their true numbers are underestimated.

## Evidence for B1 Cells in Human beings

In human beings, the identification of B1 cells has proven to be a contentious issue (89–94). Part of the reason for this relates to the difficulty in accessing sites such as the peritoneal cavity, where B1 cells are enriched in mice. Nonetheless, currently, there is limited evidence for an exact replication of the murine phenotype for B1 cells in human beings, particularly for B1a cells (89, 91, 95, 96). One report has identified a rare population of B-cells in patients with chronic variable immunodeficiency disease (97) and cells in other non-human primates (NHP) share common B1b markers (98). B-cells in human fetal cord blood and adult blood with a CD20^+^CD27^+^CD43^+^CD70^-^ phenotype, but with a variable expression of CD5, CD86, IgM, and IgD have been identified as human B1 cells (89). The definition of these cells is not solely based on surface markers as these cells also had other properties associated with B1 responses. These include (i) the ability to spontaneously secrete IgM antibody to antigens associated with B1 responses such as phosphorylcholine, a known target of B1a-derived antibody in mice and (ii) the ability to readily interact and prime T-cells. Interestingly, the probability of a human B1 cell having either or both properties correlates to its expression of CD11b. Spontaneous IgM secretion is more associated with CD11b^−^ cells and T-cell modulating activity associated with CD11b^+^ human B1 cells. Nevertheless, others have suggested that CD27^+^CD43^+^CD70^-^ B-cells may reflect a pre-plasmablast differentiation state of cells present in the blood (93). It is tempting to speculate how cumulative exposure to antigen may influence the phenotype and frequency of B1 cells in different species since B1 cells are more readily detectable in animals kept in controlled environmental conditions. Therefore, currently, there is not a universally accepted definition for B1 cells in human beings as there is for mice.

## Mice Contain Multiple B1 Subsets

B1 cells can have phagocytic and antigen-presenting functions, secrete cytokines to modulate the host and produce antibody, with these properties not necessarily mutually exclusive within individual cells (32, 99–101). Most work on B1 cells in mice has focused on B1a cells, and their activities have been well reviewed by a number of authors (see above). B1a cells express CD5 and, at least in the steady state, are the dominant B1 subset in the peritoneal cavity. They play a major role in maintaining host immunological tone and homeostasis, but also make striking contributions to the control of infections, such as influenza (15, 31, 102, 103). What is striking about the role of B1a response to influenza is that they contribute antibody through two pathways. The first is through the production of natural antibody, which is generated independent of the presence of cognate antigen. The second is the generation of antibody during infection. Influenza infection results in the accumulation of B1a cells to sites of infection and their production of antibody specific to the pathogen. B1a cells are able to generate IgM and IgA antibodies of modest affinity that can be poly-reactive. Features of the B1a response are atypical since it does not necessarily require the differentiation of B1a cells into CD138-expressing plasma cells or the loss of B1a cell markers, and the majority of the B1a antibody induced is not specific to influenza (104). This suggests a robust early and innate, if “blind” antibody response, which functions to limit infection. While this may simply be a “panic” response to a pathogen, it is also possible that these cells also modulate immunity through phagocytic or contact-dependent mechanisms.

The reported ability of B1a cells to secrete antibody after antigen exposure, but without differentiation into plasma cells, raises questions regarding the nature of antibody-secreting cells. This has only been addressed in any detail for CD5+ B1a cells, and is also an area where reports do not necessarily converge. Based on *in vitro, ex vivo*, and adoptive transfer studies, Rothstein and colleagues have shown that antibody-secreting B1a cells in the peritoneal cavity show limited expression of genes associated with plasma cells, such as blimp-1 and CD138 (105). Furthermore, IRF4-deficient peritoneal B1 cells can still secrete IgM, whereas splenic B1 cells do not (106). Nevertheless, experiments using Blimp-1 reporter mice failed to support these findings, although Blimp-1-deficient mice retain the capacity to generate some immunoglobulin (107, 108). The differences in these studies may reflect the different approaches taken or other factors. What is lacking is a detailed comparison between plasma cells generated from B-cells of different origins that compare more than the antibody isotype they produce or antigens they initially responded to, although this is clearly a technical challenge.

## B1b Cells Make Important Contributions to Protective Immunity in Mice

Much of the work examining the activities of B1a cells have focused on their role in autoimmunity, while their roles in controlling infection are less studied. B1a cells do not necessarily function in isolation and, for instance, they can collaborate with B1b cells to combat pneumococcal infections (80). Despite sharing many phenotypic features with B1a cells, such as the generation of natural antibody to self-antigens (109), there are key differences between B1a and B1b cells. There is evidence that each of these cell types has a different developmental pathway and infection may increase a progenitor population that has the potential to generate progeny with a B1a-like phenotype (26–28, 30, 31, 33, 110–116). Another difference between B1a and B1b cells is that the latter tend to exhibit a greater level of junctional diversity compared to B1a cells suggesting a broader repertoire of antigens is recognized by these cells, although this may be a more complex picture than previously envisaged (117–120). This then leads to a key question. What antigens are recognized by B1b cells and are they clinically relevant?

The first studies showing a potential clinical benefit of B1b responses came from experiments using a murine model of relapsing fever caused by the spirochete *Borrelia hermsii* (121, 122). This bacterial infection in mice results in a pronounced bacteremia, with bacterial numbers reaching >10^6–7^ bacteria/ml blood, but there is much less colonization of tissues by bacteria (123). A key reason for this is the high degree of antigenic variation in certain bacterial surface proteins (124). The infection relapses because antibody develops to the dominant clone and as this is controlled it enables the outgrowth of an antigenically distinct minor clone, which goes on to cause another episode of fever. This can occur multiple times, but typically each further round of bacteremia is less severe than the one that preceded it. This suggests there are protective antigens present that are unrelated to the variable proteins and that the antibody response to these protective antigens develops at a slower rate, due to potential reasons such as antigen density or epitope availability. IgG can contribute to protection in this model but an absolute requirement for IgM has been demonstrated (121–123). A role for B1b cells in protection against *B. hermsii* was identified after infection of a range of genetically altered mice (IL7−/− mice that are deficient in follicular B-cells), splenectomized mice, and B-cell chimeric mice (121). Protection was TI and reinfection of T-cell-deficient mice revealed that a TI memory response was induced. Significantly, B1b chimeras generated by transfer of B1b cells from convalescing mice into Rag1-deficient hosts were more efficient at controlling infection than equivalent chimeras generated using naïve B1b cells. This finding of TI memory was reproduced in a separate model involving immunization with a polysaccharide antigen from *Enterobacter cloacae* (125).

The unequivocal demonstration of a role for B1b cells in controlling this infection raised questions regarding which antigen was the target for this protective antibody. The obvious candidates were the variable surface proteins from this pathogen, which contribute to immune evasion, but this turned out not to be the case. The target of the protective B1b antibody was a conserved protein, the Factor H binding protein (fHbp), and the response to this protein could develop in the absence of T-cells (126). Antibody to fHbp accumulates slowly with time and protection is only achieved when levels of antibody to this antigen reaches a certain level. This is sufficient to explain why each round of bacteremia tends to be less severe than the one before. This is an important finding and the concept it identifies has far reaching implications for understanding protective immunity during natural infection and for vaccinology. It means that targeting cell wall localized immunodominant antigens is not necessarily required to generate immunity to different strains of the same organism. There are manifold forms of fHbp produced by different bacterial genera and these show significant variability at the protein level. This protein is of interest to vaccinologists as it is one of the protective antigens included in the Bexsero vaccine used against group B meningococcus (127). Alugupalli and colleagues extended their findings on fHbp from *B. hermsii* by generating mice with a humanized immune system, so that all B-cells are derived from human progenitors (128). They showed these chimeras generated IgM antibody to *B. hermsii* and to fHbp and that protection was dependent upon B-cells. Although not formally shown, the antibody response in these chimeras is likely to have been induced in a TI manner. This is a landmark study since it demonstrates that human B-cells are capable of generating anti-protein antibody responses in a manner that resembles the process in mice.

Parallels can be observed between B1b cell responses in mice and the B-cell response in other species to TI–II antigens, such as capsular polysaccharides (78, 80). Classically, splenic MZ B-cells are associated with the generation of antibody to such antigens, in part because of the poor responses to capsular polysaccharide vaccines observed in asplenic adults and infants, who lack a mature MZ B-cell compartment (74, 129). Nevertheless, mice deficient in MZ B-cells can still make robust responses to TI–II antigens (78, 130) so this does not negate the potential for B1b cells to be involved in such responses. Also, B1b cells are detected in the spleen after immunization with TI–II antigens and they can contribute directly or through interactions with MZ B-cells (131, 132). Although B1b cells can recognize haptenated Ficoll, the antigen itself is not a natural antigen and it is typically absent from the environment meaning that in the absence of immunization, animals are naïve to it. This has been exploited to identify the presence of cells that phenotypically and functionally resemble B1 (both B1a and B1b cells) in NHP (African green monkeys and cynomolgus monkeys) (98). NHP B1 cells have a similar phenotype to cells in mice and are CD11b^hi^ forward scatter hi, CD21^lo/−^ and CD19hi and can upregulate CD27 upon antigen encounter. Like B1b cells, in mice, they respond to TNP-Ficoll and produce IgM and IgG but little IgA.

In an earlier pivotal study, Haas and colleagues demonstrated that capsular polysaccharide from *Streptococcus pneumoniae* is a B1b antigen and that B1b-derived antibody was sufficient to protect against infection (80). Purified pneumococcal polysaccharide is currently used as a vaccine in human beings against these infections and such responses can be long lived (133, 134). The response in B-cells also differs depending whether the vaccine is conjugated or not (135). Additional studies using α-1,3 dextran from the Gram-negative bacterium *E. cloacae* have identified B1b cells as the principal reservoir of memory to bacterial polysaccharide antigens (125). Indeed, B1b cells maintain the potential to respond to pneumococcal polysaccharide throughout life (136), with the mechanisms underlying the responsiveness of B1b cells under active investigation (137). Therefore, identifying targets of B1b cells may help in developing strategies to improve vaccination against pneumococcus in the elderly, who are disproportionately susceptible to these infections and in whom vaccination with pneumovax has not had a dramatic effect on the incidence of infection (138). It is likely that the number of infections where B1b responses are important will expand. For instance, putative evidence suggests that the characteristic response of the organism *Ehrlichia muris* at least in part involves B1b cells (40, 139). Collectively, studies such as those highlighted above show that B1b antigens are present in a plethora of bacterial genera of major clinical importance and that these antigens can be key targets of protective immunity.

## *Salmonella* Infection as a Model to Study B1b Cells

**Figure 2:**
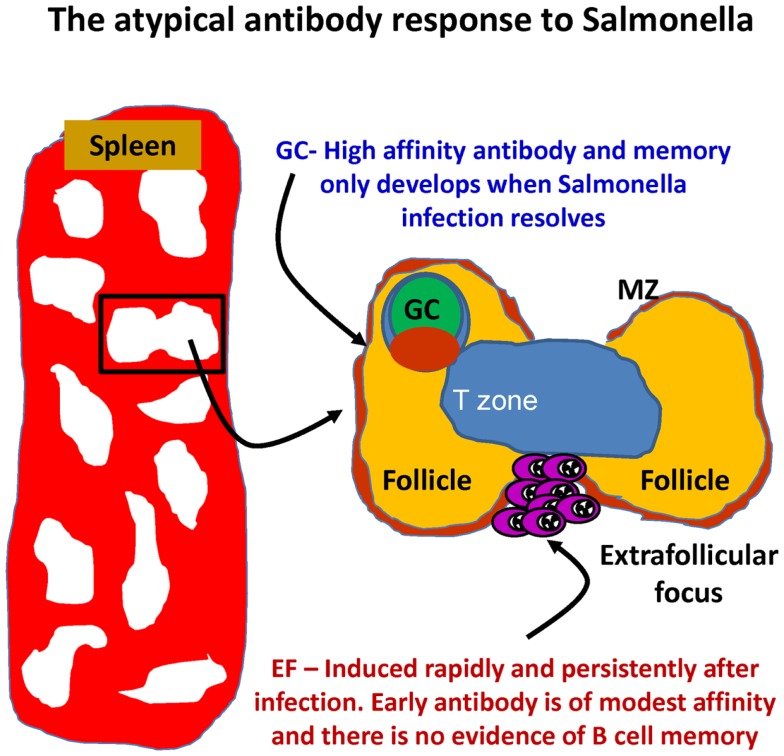
**The development of antibody responses in mice after *Salmonella* infection**. After infection, there is a rapid induction of extrafollicular responses, so that IgM and IgG plasma cells are readily detectable by day 3. These events occur in the absence of germinal center (GC) responses that are absent until infection has all but cleared. MZ – marginal zone.

In murine systems, B1b cells can make a significant contribution to protection. Our own work has examined the B-cell response in the Gram-negative bacterium *Salmonella enterica* serovar Typhimurium (49). Clearance of primary *S*. Typhimurium infections in mice requires an intact and persisting CD4 T-cell response regulated by the transcription factor T-bet (140–144). Antibody can protect against secondary infection or after immunization (46, 81, 145, 146). We came to study *S*. Typhimurium indirectly as a development of our use of model antigens such as ovalbumin and the bacterial TLR5 ligand flagellin (43, 147). Using these antigens, we could show how altering the context in which an antigen was encountered could significantly alter the T- and B-cell response to it. Through immunohistological examination of the splenic plasma-cell response after systemic *S*. Typhimurium infection, we noted that the response was highly atypical (Figure 2). In particular, we noted that the bacterium induced a rapid EF plasmablast response (49). On day 3 after *S*. Typhimurium, the majority of IgM plasmablasts in the red pulp were in cell cycle, and their numbers had already increased >10-fold. In parallel, on day 3, there was also a 3–4-fold increase in IgG2a-switched plasmablast numbers, rising to around 20-fold higher by day 4, when most IgG2a cells are in cell cycle. In contrast to EF responses after immunization with alum-precipitated proteins (148), the induction of the IgM response was CD40L-independent, whereas switching was CD40L dependent [(49); data not shown]. Atypically, the EF IgM and IgG response in WT mice occurred in the absence of a detectable GC response, which was not observed until the infection was largely cleared (around day 35 in the described model). Therefore, for at least 3 weeks, this model allows the assessment of an EF response in the absence of a confounding influence from GC.

The speed and extent of the EF response and the T-cell independence of its induction had two major implications. First is that there is a significant availability of antigen for B-cells to access, despite the organism having an intracellular life-style and only inducing a low grade bacteremia. Second, it suggests there is likely to be a significant precursor B-cell pool that is enriched for antigens present within this bacterium. Identifying which antigens were targets of the atypical B-cell response was greatly aided by the characterization of the prolonged antibody response to the major porin proteins from *S*. Typhi OmpF and OmpC. Work by Lopez-Macias, Isibasi and colleagues showed that immunization of human beings and mice with purified, soluble porins induced long-lived bactericidal antibody against typhoid (149, 150). These proteins were good candidates as targets of the EF antibody response in that they naturally share features of TI–II antigens such as existing as oligomers and thereby containing repeating epitopes (151). Immunization with purified porin proteins from *S*. Typhimurium showed that these proteins induced a TI response and induced protective antibody primarily through the presence of an additional porin, OmpD, which is absent in *S*. Typhi (46). Characterizing the peritoneal B-cell response to porin proteins and live *S*. Typhimurium showed that both antigens induced a B1b cell response. In contrast, heat-killed bacteria or purified, monomeric flagellin did not. Immunization with TLR grade LPS also induces features of a B1b response, although it is unclear how much of this is antigen-specific or induced through its mitogenic effects. In responses to other Gram-negative bacteria, LPS may induce a B1a response (152). A marker commonly used to help identify B1b cells is CD11b. After infection of WT, but not T-cell-deficient mice, the majority of B1b cells was not CD11b+. Thus, it is possible that examination of the response in WT mice using CD11b may under-estimate the numbers of B1b cells, although other reasons may help explain this, such as the maturity of the B1b cells after infection or that additional B1 lineages are recruited to the response.

This model allowed the investigation of other key questions regarding B1b cells, one of which is whether multiple B1b antigens exist within the same species. This was investigated using the Vi antigen from *S*. Typhi. This antigen, in purified form, is used as a vaccine against typhoid in human beings (146). Since *S*. Typhi is a human pathogen with limited infectivity in non-primates, it was necessary to examine the response in mice to *S*. Typhimurium engineered to express Vi antigen and to purified Vi itself (81, 153). This allowed a combined approach to be used where the response to Vi in the context of the bacterium or after immunization with the purified antigen could be examined. These experiments confirmed the TI nature of the response to Vi and showed that it induced a B1b cell response. Furthermore, the antibody generated by B1b chimeras was sufficient to provide protection against challenge with *S*. Typhimurium expressing Vi. Thus, the same species of bacterium contains multiple B1b antigens, suggesting they may be more widely distributed than was perhaps originally perceived when the pioneering studies were being undertaken.

## Conclusion

The evidence clearly shows that B1 cells play an important role in providing antibody against infection. Between the B1 subsets, B1b cells are most associated with providing responsive antibody during natural infection or after vaccination. The targets of B1b cell-derived antibody appear to have a disproportionate likelihood of also being protective antigens and many of these antigens are known to induce protective responses in human beings. This strongly supports the concept that the B-cell receptor usage by B1b cells is not random. Furthermore, the antigens recognized by B1b cells are not limited to any one genus or Gram classification. The detection of multiple protective B1b antigens in a single species suggests that B1b antigens are widespread. Therefore, even allowing for the incomplete understanding of B1 responses in human beings examining B1b antibody responses in mice is likely to be a rewarding avenue for identifying putative vaccine candidates.

**Figure 3:**
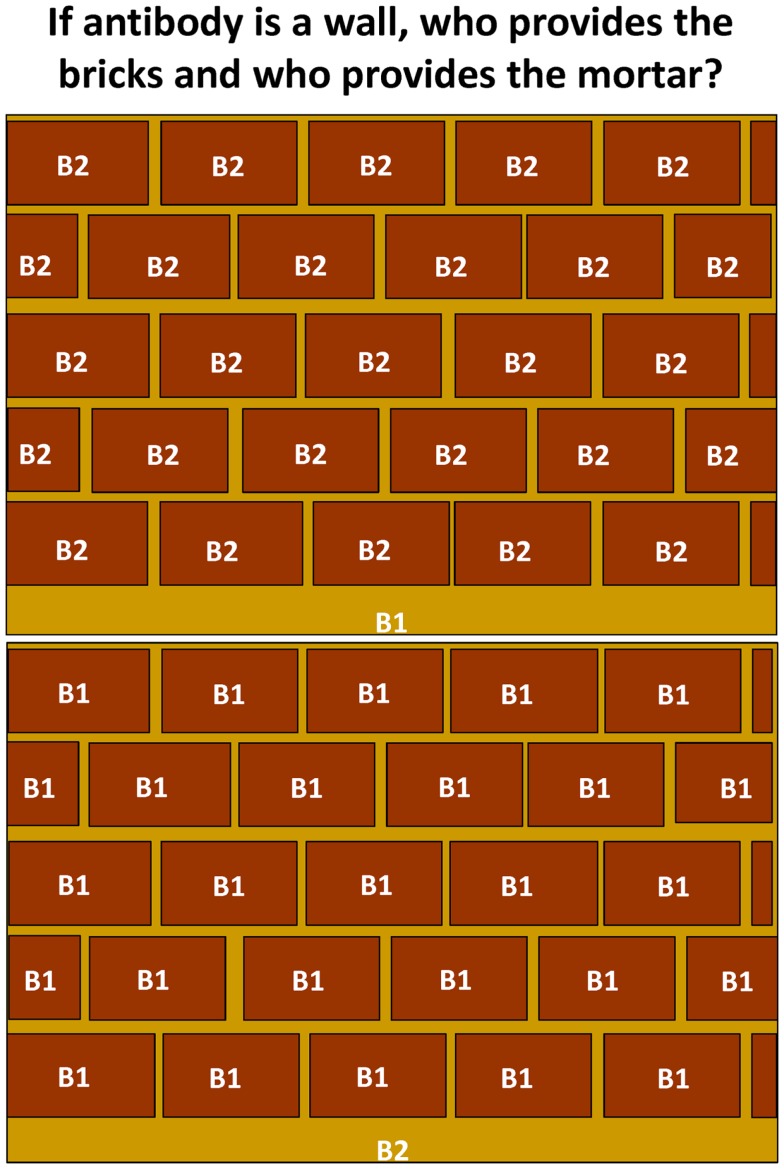
**The relative contribution of antibody from B1 and B2 cells**. Antibody provides a wall of protection against infection. Antibody to many B1(b) antigens is also protective and suggests that a wide repertoire of responses are not always necessary to protect against infection. Since antibody to many antigens is not protective, it may be that antibody derived from B1 cells provides greater protection in relative terms than that from B2 cells, particularly follicular B-cells. In the context of this figure, the mortar fills the gaps between the bricks, which provide the majority of protective coverage.

From a personal perspective, there remain a number of unresolved questions regarding the contribution of antibody from and the biology of B1 cells. How complete is our understanding of the nature of selection of B1 cells? How can they be selectively enriched to recognize protective antigens and epitopes? How widespread are B1b cells in human beings? How great is the contribution of antibody from B1b cells to protection after vaccination or natural infection compared to B2 cells and do B1b cells primarily target bacteria and is this restricted to cell wall antigens? Thus, if we think of the protection provided by antibody as a wall then most of a wall is occupied by the bricks, with the gaps between filled by mortar. If antibody to B1b antigens is sufficient to protect against most infections, then the coverage offered by this would be sufficient to protect against the majority of bacterial threats (Figure 3). The remaining spaces would be filled by antibodies derived from other B-cell subsets and contribute to protection against antigens such as soluble toxins where there is no evidence of any contribution of B1b cells. How does conjugating a B1b antigen, such as pneumococcal polysaccharide to a typical protein carrier like CRM197, alter the responsiveness of B1b cells to this antigen? Furthermore, how well can B1b cells switch? Experiments using *Salmonella* show how closely the TI induction of the response and the TD switching in the same response are coupled and so we suspect they are two features of the same B-cell population. Is this really the case or are there two parallel responses induced concurrently? Finally, can B1b cells take part in the GC response? The expansion in the numbers and types of antigens recognized by these cells, the models available, and the intensive investigations underway in human beings make the coming years an exciting time for this field and have implications for both basic and applied immunology, microbiology, and vaccinology.

## Conflict of Interest Statement

Adam F. Cunningham has a patent on OmpD – Pub. No.: WO/2010/029293 International Application No.: PCT/GB2009/002159: Non-typhoidal *Salmonella* vaccines (http://patentscope.wipo.int/search/en/WO2010029293). Granted and currently under license with NVGH and held in South Africa, China, Brazil, Canada, US, and UK. The other co-authors declare that the research was conducted in the absence of any commercial or financial relationships that could be construed as a potential conflict of interest.
